# Malaria-associated risk factors among adolescents living in areas with persistent transmission in Senegal: a case–control study

**DOI:** 10.1186/s12936-022-04212-8

**Published:** 2022-06-20

**Authors:** Fassiatou Tairou, Abdoulaye Diallo, Ousmane Sy, Aminatou Kone, Isaac Akhenaton Manga, Khadim Sylla, Souleye Lelo, Cheikh Binetou Fall, Doudou Sow, Magatte Ndiaye, Babacar Faye, Roger C. K. Tine

**Affiliations:** 1 Department of Medical Parasitology, University Cheikh Anta Diop of Dakar, Dakar, Senegal; 2 Ministry of Health and Social Action, Dakar, Senegal; 3 Malaria Research and Training Center, University of Bamako, Bamako, Mali

**Keywords:** Malaria, Transmission, Epidemiology, Adolescent, Senegal

## Abstract

**Background:**

In Senegal, malaria morbidity has sharply decreased over these past years. However, malaria epidemiology remains heterogeneous with persistent transmission in the southeastern part of the country and many cases among older children and adolescents. Little is known about factors associated with clinical malaria among this group. A better understanding of malaria transmission among this newly emerging vulnerable group will guide future interventions targeting this population group. This study aimed to identify factors associated with clinical malaria among adolescents in Senegal.

**Methods:**

A case–control study was conducted from November to December 2020 in four health posts located in the Saraya district. Cases were defined as adolescents (10–19 years) with an uncomplicated malaria episode with fever (temperature > 37.5°) or a history of fever and positive malaria rapid diagnostic test (RDT). Controls were from the same age group, living in the neighbourhood of the case, presenting a negative RDT. A standardized, pre-tested questionnaire was administered to each study participant followed by a home visit to assess the participant's living conditions. Factors associated with clinical malaria were assessed using stepwise logistic regression analysis.

**Results:**

In total, 492 individuals were recruited (246 cases and 246 controls). In a multivariate analysis, factors associated with clinical malaria included non-use of long-lasting insecticidal net (LLIN) (aOR = 2.65; 95% CI 1.58–4.45), non-use of other preventive measures (aOR = 2.51; 95% CI 1.53–4.11) and indoor sleeping (aOR = 3.22; 95% CI 1.66–6.23). Protective factors included 15–19 years of age (aOR = 0.38; 95% CI 0.23–0.62), absence of stagnant water around the house (aOR = 0.27; 95% CI 0.16–0.44), having a female as head of household (aOR = 0.47; 95% CI 0.25–0.90), occupation such as apprentice (OR = 0.24; 95% CI 0.11–0.52).

**Conclusions:**

The study revealed that environmental factors and non-use of malaria preventive measures are the main determinants of malaria transmission among adolescents living in areas with persistent malaria transmission in Senegal. Strategies aimed at improving disease awareness and access to healthcare interventions, such as LLINs, are needed to improve malaria control and prevention among these vulnerable groups.

## Background

Malaria remains a major public health problem globally and particularly in sub-Saharan Africa, despite the substantial decrease of malaria burden over these past 15 years, due to the scale-up of standard control interventions such as prompt diagnosis and treatment, long-lasting insecticidal nets (LLINs), and indoor residual spraying (IRS) [1]. In 2020, 241 million malaria cases were reported in 85 endemic countries, with an estimate of 228 million cases representing 95% of cases, in sub-Saharan Africa (SSA). In addition, 627,000 deaths were attributed to malaria globally in 2020, among which, 602,000 occurred in SSA [2].

In Senegal, malaria is endemic and unevenly distributed in the 14 regions of the country. In 2019, by the time of the current study initiation, the National Malaria Control Programme (NMCP) reported 354,708 malaria cases representing an incidence of 21.9 per 1000. Most of these cases (81%) were reported in three regions located in the southeastern part of the country: Kolda, Tambacounda and Kédougou [3]. Current malaria policies in Senegal are mainly focused on achieving universal coverage of effective interventions, including malaria diagnosis, predominantly through rapid diagnostic tests (RDTs); treatment of all malaria cases with artemisinin-based combination therapy (ACT); intermittent preventive treatment for pregnant women (IPTp); LLINs; and/or IRS [4, 5]. Seasonal malaria chemoprevention and reactive case detection have recently been introduced in specific geographic areas in Senegal [6, 7]. Senegal adopted a strategy of universal coverage of LLINs in 2010. Since then, LLINs have been rolled out through mass campaigns every 3 years, in parallel with a routine distribution system at health facility level, targeting mainly pregnant women during antenatal care visits, and children under 5 years old. From 2013 to 2017, proportional morbidity and mortality nationwide decreased from 5.4 to 3.3% and from 7.5 to 1.7%, respectively. However, these indicators increased from 2017 to 2018, with proportional morbidity increasing from 3.3 to 4.8% and proportional mortality from 1.7 to 3.5% [8]. From 2018 to 2019, these indicators have decreased again, proportional morbidity dropping from 4.8 to 3.0% and mortality from 3.5 to 1.7% [3]. In addition, longitudinal routine malaria data from the NMCP showed that over the past 5 years, the majority of malaria cases occurred among 10–15 years-old adolescents (Tine R, pers. comm.).

A study conducted in Dielmo in the central part of Senegal, to assess the evolution of malaria morbidity in adults before and after the implementation of LLINs, showed that 15–19 years-old adolescents were the most vulnerable group during malaria upsurges [9]. Recent studies conducted in SSA countries have shown a higher prevalence of malaria among older children and adolescents [10–12], or an increased risk of malaria among these groups [13–16]. This could be explained by several reasons including the lack of optimal usage of LLINs [17, 18] and sub-optimal behaviour such as staying outdoors during the night, which corresponds to the time of malaria vector activity [18, 19]. Despite these trends, children under 5 years of age and pregnant women are still considered the most at-risk groups for malaria in Senegal and consequently represent the usual target of the majority of available control measures such as IPTp and routine distribution of LLINs. This may lead to a residual human malaria reservoir with adolescents and older children who are not the primary target of control measures. There is thus an urgent need to adapt current malaria control strategies to account for emerging of additional vulnerable groups such as older children and adolescents. A better understanding of malaria-associated risk factors and their determinants among older children and adolescents is needed to optimize malaria control among these specific key populations. But there is a lack of evidence about factors associated with clinical malaria among this age group. Studies are needed to fill these scientific gaps that may hamper malaria control and elimination efforts.

This case–control study was conducted to assess factors associated with clinical malaria among adolescents to inform future control strategies among key vulnerable populations living in areas with persistent malaria transmission in Senegal.

## Methods

### Study setting

The study was conducted in the health district of Saraya, located in the Kedougou region in the southeastern part of Senegal, 800 km from Dakar, the capital. The district shares a border with Mali at the east, Guinea in the south, the region of Tambacounda in the north, and the health district of Kedougou in the west. The health district occupies a land area of 6837 sq km. The population is composed of the rural communities of Bembou, Medina Baffe, Sabadola, Khossanto, and Missirah Sirimana. The district has one health centre, 22 health posts, 28 health huts, and 78 villages each with a village volunteer for malaria case management (*Dispensateur de soins à domicile (*DSDOM)). The climate is Sudano-Guinean with a dry season and a rainy season. Malaria is meso-endemic and stable in Saraya, with a long transmission season lasting four to six months from July to December. Transmission intensity remains high with 20 to 100 infectious bites/person/year and high morbidity during the transmission period. The major vectors are *Anopheles gambiae, Anopheles arabiensis *sensu lato (*s.l*.)*, Anopheles funestus,* and *Anopheles nili* [20]. Malaria incidence was 376.6 per 1000 in 2017, 487.2 per 1000 in 2018, 379.8 per 1000 in 2019 [3], and 607.4 per 1000 in 2020 [5].

### Study design and participants

A case–control study was conducted from November to December 2020 in four health posts (Bembou, Diakhaling, Khossanto, Sambrambougou), selected purposively based on reported malaria morbidity in 2019 and community accessibility during the rainy season. The number of confirmed malaria cases in 2019 was 1404 in Bembou, 1485 in Diakhaling, 2166 in Khossanto, and 3311 in Sambrambougou.

Cases were recruited from participating health posts while the controls were recruited within the same community, in the same village as the case but not in the same house (case and control houses were distant from each other by at least two other houses). A case was defined as an adolescent (10–19 years old) coming to seek care at one of the participating health posts for uncomplicated malaria episode defined as fever (temperature > 37.5°) or history of fever within the previous 48 h, with positive malaria RDT according to the NMCP guidelines. Control was a person of the same age group, living in the neighbourhood of the case, with negative RDT.

Non-inclusion criteria for both, the cases and the controls included: (i) children under 10 years old; (ii) individual who does not live in the study area; (iii) subject who received anti-malarial treatment 3 weeks before the study.

### Data collection methods

An electronic data collection platform was used. Data were collected using android tablets with the electronic questionnaire developed on REDCap, which is a Research Electronic Data Capture software compatible with android technology [21, 22]. Data collected from the tablets were then synced via an internet connection to a server hosted at University Cheikh Anta Diop in Dakar for storage. Data were extracted from the server for cleaning and analysis. Before the start of the study, nurses and community health workers in participating health posts were trained on the study procedures, including informed consent and administration of the questionnaire. For each participant who gave informed consent, an electronic standardized questionnaire was administered to collect data on socio-demographic characteristics of the participant and those of the household head, the household assets (water source, type of toilet, ownership of certain items such as television, radio, fridge, etc.), ownership and use of LLIN, use of other malaria control measures, individual behaviour during the mosquito ‘biting time’ (stay outdoors at evening/night, sleeping outdoors during the night). Home visits were performed for both cases and controls to assess the living conditions of the participants (types of wall, floor, roof), as well as the environmental factors such as stagnant water within and in the vicinity of the household, overgrown vegetation/bushes inside and in the vicinity of the houses of the participants.

### Statistical methods

#### Sample size calculation

For the sample size calculation, the use of LLINs was considered the main determinant associated with malaria. The proportion of LLIN use among children in the study area is 62% [23]; assuming a risk alpha at 5%, with a ratio of 1 control for 1 case, the study was powered at 80% to detect an odds ratio of 0.6 if 492 individuals were recruited (246 cases *vs* 246 controls).

#### Data analysis

Data were extracted from the server and analysed using STATA software [24]. A composite variable of wealth index was estimated, based on the assets of the households [25]. Housing variables are not included in the wealth index calculation as in the index used in the Demographic and Health Survey (DHS) [23] to avoid correlation between variables. Impact of housing materials on malaria risk as shown in other studies are assessed separately [25, 26]. The index was then categorized into five wealth quintiles (richest, rich, middle, poor, poorest) using the Principal Component Analysis method (PCA) [23, 27]. Variables characterizing household materials, including the type of wall, roof and floor were grouped according to the DHS definition [23] as traditional (rudimentary and natural) and modern to create a binary housing variable. Modern houses were then defined as those that have a modern wall, modern roof and modern floor; while traditional houses were those that have a rudimentary and natural wall, floor and roof [25]. Modern roof materials include cement/concrete, wood planks, tile, metal, while traditional roof materials include thatch/straw, bamboo. Modern floor materials include cement and ceramic tiles whereas traditional floor materials include earth/mud and bamboo. Modern wall materials consisted of cement and wood/planks while traditional wall materials consisted of earth/mud and bamboo/palm.

The percentage was used to assess the frequency of each outcome with a 95% confidence interval (CI). Characteristics of all participants included in the study were tabulated. Proportions were compared using the Chi-square test or Fisher exact test where appropriate (univariate analysis).

Factors associated with malaria were assessed using a logistic regression model. Covariates with a p-value < 0.20 in univariate analysis were introduced in the multivariate model. From the final model, adjusted odds ratios were derived with their 95% CI. Model validity was tested using the Hosmer–Lemeshow goodness of fit test. The performance of the final model was assessed by the area under the curve, and Akaike and Bayesian information criteria. In addition, a test for multicollinearity between variables was done using the variance inflation factor. The significance level of the different tests was 0.05, two-sided.

### Ethical considerations

The study protocol was approved by the University Cheikh Anta Diop Institutional Review Board (CER/UCAD/AD/MSN /039/2020). Before the start of the study, administrative authorization was sought from the regional and district medical authorities in Kedougou and Saraya. In the field, participation in the study was strictly voluntary. Before any enrolment, written informed consent was obtained from parents or caregivers for adolescents under 18 years old, while 18–19 years-old individuals were invited to consent themselves. In addition, a child assent was sought from 15 to 17 years-old participants. A unique identification code was attributed to each participant. Personal identified data collected for the household head were de-identified before the data extraction. All analyses were performed using participant's identification code to ensure maximum confidentiality. Access to the study data was restricted and information collected was only used for the study purpose.

## Results

### Participants’ characteristics at enrolment

#### Demographics

A total of 492 individuals were recruited (246 cases and 246 controls). Cases and controls were similar in terms of demographic characteristics. There was no statistical difference between cases and controls (Table 1).

**Table 1:** Socio-demographic characteristics of the participants and the household heads

	Case (246)		Control (246)		P value
	Number (%)	95% CI	Number (%)	95% CI	
*Characteristics of participants*					
Age group					
10–14 years	130 (52.85%)	(46.49–59.22%)	106 (43.09%)	(36.81–49.53%)	0.1356
15–19 years	116 (47.15%)	(40.78–53.60%)	140 (56.91%)	(50.47–63.19%)	0.1196
Gender					
Male	135 (54.88%)	(48.43–61.21%)	136 (55.28%)	(48.84–61.60%)	0.9472
Female	111 (45.12%)	(38.79–51.57%)	110 (44.72%)	(38.40–51.16%)	0.9523
Ethnicity					
Pular	46 (18.70%)	(14.03–24.14%)	43 (17.48%)	(12.95–22.81%)	0.8813
Malinke	171 (69.51%)	(63.35–75.20%)	181 (73.58%)	(67.60–78.98%)	0.3973
Bambara	17 (6.91%)	(4.08–10.83%)	8 (3.25%)	(1.41–6.30%)	0.7136
Others^‡^	12 (4.88%)	(2.54–8.37%)	14 (5.69%)	(3.15–9.36%)	0.9269
Occupation					
Student	161 (65.45%)	(59.14–71.37%)	134 (54.47%)	(48.02–60.81%)	0.0548
Maid	46 (18.70%)	(14.03–24.14%)	35 (14.23%)	(10.11–19.23%)	0.5937
Apprentice*	13 (5.28%)	(2.84–8.87%)	48 (19.51%)	(14.75–25.02%)	0.2199
Gold digger	18 (7.32%)	(4.39–11.32%)	13 (5.28%)	(2.84–8.87%)	0.8197
Others†	8 (3.25%)	(1.41–6.31%)	16 (6.50%)	(3.76–10.35%)	0.7402
*Characteristics of household head*					
Age					
23–33	26 (10,57%)	(7.02–15.10%)	27 (10,98%)	(7.36–15.57%)	0.9616
34–43	57 (23,17%)	(18.05–28.95%)	61 (24,80%)	(19.53–30.68%)	0.8359
44–53	74 (30,08%)	(24.42–36.23%)	55 (22,36%)	(17.31–28.09%)	0.3275
54–63	52 (21,14%)	(16.20–26.78%)	79 (32,11%)	(26.32–38.34%)	0.1701
64–87	16 (6,50%)	(3.76–10.35%)	24 (9,76%)	(6.35–14.17%)	0.7166
Missing	21 (8,54%)	(5.36–12.75%)	0 (0,00%)	-	
Gender					
Male	222 (90,24%)	(85.83–93.65%)	209 (84,96%)	(79.87–89.18%)	0.0956
Female	24 (9,76%)	(6.35–14.17%)	37 (15,04%)	(10.82–20.13%)	0.5487
Education level					
None	79 (32.11%)	(26.32–38.34%)	74 (30,08%)	(24.42–36.23%)	0.7864
Koranic school	75 (30.49%)	(24.80–36.65%)	86 (34,96%)	(29.01–41.28%)	0.5470
Primary	69 (28.05%)	(22.53–34.11%)	62 (25,20%)	(19.90–31.11%)	0.7128
Secondary	20 (8.13%)	(5.04–12.28%)	20 (8,13%)	(5.04–12.28%)	1.0000
University	3 (1.22%)	(0.25–3.52%)	4 (1,63%)	(0.44–4.11%)	0.9642
Matrimonial status					
Single	7 (2.85%)	(1.15–5.78%)	8 (3.25%)	(1.41–6.31%)	0.9642
Married	237 (96.34%)	(93.17–98.31%)	229 (93.09%)	(89.17–95.92%)	0.1160
Other	2 (0.81%)	(0.10–2.91%)	9 (3.66%)	(1.69–6.83%)	0.8345
Wealth index					
Poorest	61 (24,80%)	(19.53–30.68%)	37 (15,04%)	(10.82–20.13%)	0.2511
Poor	44 (17,89%)	(13.31–23.26%)	55 (22,36%)	(17.31–28.09%)	0.5832
Medium	48 (19,51%)	(14.75–25.02%)	50 (20,33%)	(15.48–25.90%)	0.9191
Rich	49 (19,92%)	(15.11–25.46%)	50 (20,33%)	(15.48–25.90%)	0.9594
Richest	44 (17,89%)	(13.31–23.26%)	54 (21,95%)	(16.94–27.65%)	0.6181

^*^Apprentice includes tailor, driver, mechanic, mason apprentices

^‡^Other ethnicity include Diakhakhe, Dialouke, Mossi, Serere, Sarakhole, Wolof, Bassari

^†^Other occupation include farmer, taxi driver, seller and talibe (children in placement in Koranic school)

### Participants’ living conditions, LLIN ownership and usage

Most of the cases (81.71%) and the controls (93.09%) reported LLIN ownership but LLIN ownership was significantly higher among controls (p = 0.0003). The proportion of cases that reported the use of LLIN was 63.82% while that among controls was 82.52% (p = 0.0001). LLIN usage the previous night was significantly higher among controls (78.46%) compared to cases (58.94%) (p = 0.0001). Almost all the controls (92.68%) reported sleeping outdoors sometimes, compared to the cases (72.76%) (p < 10^–3^). Stagnant water in the vicinity of the household was observed for 46.75% of the cases and 22.76% of the controls (p = 0.0025). The type of floor of the house was traditional for 80.49% of the control compared to 58.13% for the cases (p < 10^–3^) (Table 2).

**Table 2:** Distribution of the study population according to the preventive measures and housing conditions

Variables	Case (246)		Control (246)		
	Number (%)	95% CI	Number (%)	95% CI	P value
LLIN ownership					
Yes	201 (81.71%)	(76.29–86.33%)	229 (93.09%)	(89.17–95.92%)	**0.0003**
No	45 (18.29%)	(13.67–23.70%)	17 (6.91%)	(4.08–10.83%)	0.2651
LLIN use					
Yes	157 (63.82%)	(57.47–69.83%)	203 (82.52%)	(77.19–87.05%)	**0.0001**
No	89 (36.18%)	(30.17–42.53%)	43 (17.48%)	(12.94–22.81%)	0.0281
LLIN use the previous night					
Yes	145 (58.94%)	(52.52–65.15%)	193 (78.46%)	(72.79–83.43%)	**0.0001**
No	101 (41.06%)	(34.85–47.48%)	53 (21.54%)	(16.57–27.21%)	0.0154
Use of other preventive measures^‡^					
Yes	146 (59.35%)	(52.93–65.54%)	163 (66.53%)	(60.24–72.41%)	0.1915
No	100 (40.65%)	(34.45–47.07%)	82 (33.47%)	(27.59–39.76%)	0.3193
Sleep outdoors					
Yes	179 (72.76%)	(66.74–78.23%)	228 (92.68%)	(88.68–95.61%)	**0.0000**
No	67 (27.24%)	(21.77–33.26%)	18 (7.32%)	(4.39–11.32%)	0.0747
Stay outdoors at evening/night					
Yes	245 (99.59%)	(97.76–99.99%)	244 (99.19%)	(97.09–99.90%)	0.5699
No	1 (0.41%)	(0.010–2.24%)	2 (0.81%)	(0.01–2.91%)	-
Stagnant water outside the house					
Yes	115 (46.75%)	(40.38–53.19%)	56 (22.76%)	(17.68–28.52%)	**0.0025**
No	131 (53.25%)	(46.81–59.66%)	190 (77,24%)	(71.48–82.32%)	0.0000
Stagnant water inside the house					
Yes	19 (7.72%)	(4.71–11.80%)	12 (4,88%)	(2.55–8.37%)	0.7567
No	227 (92.28%)	(88.20–95.29%)	234 (95,12%)	(91.63–97.45%)	0.2089
Grass/bushes outside the house					
Yes	144 (58.54%)	(52.10–64.76%)	126 (51,22%)	(44.79–57.62%)	0.2276
No	102 (41.46%)	(35.24–47.90%)	120 (48,78%)	(42.39–55.21%)	0.2750
Grass/bushes inside the house					
Yes	43 (17.48%)	(12.95–22.81%)	47 (19.11%)	(14.39–24.58%)	0.8418
No	203 (82.52%)	(77.19–87.05%)	199 (80.89%)	(75.42–85.61%)	0.6725
Type of roof					
Traditional	114 (46.34%)	(39.98–2.79%)	119 (48,37%)	(41.98–54.81%)	0.7564
Modern	132 (53.66%)	(47.21–60.02%)	127 (51,63%)	(45.19–58.02%)	0.7436
Type of wall					
Traditional	192 (78.05%)	(72.35–83.06%)	201 (81.71%)	(76.30–86.33%)	0.3653
Modern	54 (21.95%)	(16.94–27.65%)	45 (18.29%)	(13.67–23.70%)	0.6520
Type of floor					
Traditional	143 (58.13%)	(51.69–64.37%)	198 (80.49%)	(74.98–85.25%)	**0.0000**
Modern	103 (41.87%)	(35.63–48.31%)	48 (19.51%)	(14.75–25.02%)	0.0072
Type of house					
Traditional	205 (83.33%)	(78.08–87.77%)	218 (88.62%)	(83.97–92.30%)	0.1165
Modern	41 (16.67%)	(12.23–21.92%)	28 (11.38%)	(7.70–16.03%)	0.5403

Bold values indicate significant differences between the cases and the controls

^‡^ Other preventive measures include insecticide, smoke coil, weeding, indoor residual spraying, sewage evacuation, traditional methods

### Factors associated with malaria among the study participants

In the multivariate analysis, in overall, factors associated with clinical malaria included non-use of LLIN (aOR = 2.65; 95% CI 1.58–4.45), non-use of other preventive measures (aOR = 2.51; 95% CI 1.53–4.11) and indoor sleeping (aOR = 3.22; 95%CI 1.66–6.23). Protective factors included age range between 15–19 years (aOR = 0.38; 95% CI 0.23–0.62), absence of stagnant water around the house (aOR = 0.27; 95% CI 0.16–0.44), having a female as head of household (aOR = 0.47; 95% CI 0.25–0.90), occupation such as apprentice (OR = 0.24; 95%CI = 0.11–0.52) (Table 3).

**Table 3:** Factors associated with clinical malaria in the study participants

Parameters	Univariate analysis		Multivariate analysis	
	OR (95% CI)	P value	aOR (95% CI)	P value
Age				
10–14 years	1		1	
15–19 years	0.68 (0.47–0.96)	**0.031**	0.38 (0.23–0.62)	**0.000**
Occupation of the participant				
Student	1		1	
Maid	1.09 (0.66–1.80)	0.723	1.16 (0.60–2.24)	0.663
Apprentice^#^	0.23 (0.12–0.43)	**0.000**	0.24 (0.11–0.52)	**0.000**
Gold Digger	1.15 (0.54–2.44)	0.711	0.62 (0.23–1.66)	0.337
Others^‡^	0.36 (0.13–0.95)	**0.040**	0.50 (0.16–1.55)	0.233
LLIN use				
Yes	1		1	
No	2.19 (1.30–3.71)	**0.004**	2.65 (1.58–4.45)	**0.000**
Use of other preventive measures^†^				
Yes	1		1	
No	1.36 (0.94–1.97)	0.100	2.51 (1.53–4.11)	**0.000**
Sleep outdoors				
Yes	1		1	
No	4.74 (2.72–8.27)	**0.000**	3.22 (1.66–6.23)	**0.001**
Stagnant water outside the house				
Yes	1		1	
No	0.34 (0.22–0.50)	**0.000**	0.27 (0.16–0.44)	**0.000**
Household head gender				
Male	1		1	
Female	0.61 (0.35–1.06)	0.077	0.47 (0.25–0.90)	**0.022**

Analysis of factors associated with malaria was adjusted on the following factors: participant gender, participant age, participant occupation, household head gender, household head age, wealth index, LLIN use, use of other preventives measures, outdoor sleeping, presence of stagnant water outside the house, presence of stagnant water inside the house, presence of bushes/overgrown vegetation outside the house, type of house. The goodness of fit test: Hosmer–Lemeshow, chi^2^(7) = 12.38, p = 0.0888. BIC = − 2264.750, AIC = 1.186, AUC = 0.7640

Bold values indicate significant associations

^#^Apprentice includes tailor, driver, mechanic, mason apprentices

^‡^Other occupations include farmer, taxi driver, seller, and *talibé* (children in placement in Koranic school)

^†^Other preventives measures include insecticide, smoke coil, weeding, IRS, sewage evacuation, traditional methods

When stratified by age groups, factors associated with malaria among 10–14 years old children include non-use of LLIN (aOR = 3.36; 95% CI 1.24–9.15), non-use of other preventive measures (aOR = 3.32; 95% CI 1.61–6.85), indoor sleeping (aOR = 4.21; 95% CI 1.30–13.66). Protective factors included occupation such as apprentice (aOR = 0.07; 95% CI 0.020.30) and other occupation including children in placement in koranic school (“*talibe*”) and seller (aOR = 0.13; 95% CI 0.03–0.59); absence of stagnant water around the house (aOR = 0.26; 95% CI 0.11–0.59) and absence of stagnant water inside the house (aOR = 0.14; 95% CI 0.02–0.97) (Table 4).

**Table 4:** Factors associated with malaria among 10–14 years old adolescents

Parameters	Univariate analysis		Multivariate analysis	
	OR (95% CI)	P-value	aOR (95% CI)	P-value
Occupation of participant				
Student	1		1	
Apprentice	0.06 (0.017–0.20)	**0.000**	0.07 (0.02–0.30)	**0.000**
Others^‡^	0.12 (0.03–0.45)	**0.001**	0.13 (0.03–0.59)	**0.008**
LLIN use				
Yes	1		1	
No	4.34 (1.91–9.85)	**0.000**	3.36 (1.24–9.15)	**0.018**
Use of other preventive^†^ measures				
Yes	1		1	
No	1.92 (1.12–3.30)	**0.018**	3.32 (1.61–6.85)	**0.001**
Sleep outdoors				
Yes	1		1	
No	4.06 (1.70–9.70)	**0.002**	4.21 (1.30–13.66)	**0.017**
Stagnant water outside the house				
Yes	1		1	
No	0.23 (0.12–0.44)	**0.000**	0.26 (0.11–0.59)	**0.001**
Stagnant water inside the house				
Yes	1		1	
No	0.39 (0.10–1.48)	0.168	0.14 (0.02–0.97)	**0.046**

Analysis of factors associated with malaria was adjusted by the following variables: participant gender, participant occupation, age of the household head, wealth index, LLIN use, use of other preventive measures, outdoor sleeping, presence of stagnant water outside the house, presence of stagnant water inside the house, type of house. Goodness of fit test: Hosmer–Lemeshow, chi^2^(5) = 4.46, p = 0.4849. BIC = − 972.891, AIC = 1.089, AUC = 0.7921

Bold values indicate significant associations

^‡^Other occupation includes *talibe* and seller

^†^Disposal of wastewater, wearing of long clothes and IRS

Factors associated with malaria among 15–19 years-old adolescents include non-use of LLINs (aOR = 2.20; 95% CI 1.22–3.98), indoor sleeping (aOR = 3.79; 95% CI 1.64–8.77). Protective factors included having a female as head of household (aOR 0.16; 95% CI 0.04–0.60) and absence of stagnant water around the house (aOR 0.46; 95% CI 0.26–0.86) (Table 5).

**Table 5:** Factors associated with malaria among 15–19 years-old adolescents

Parameters	Univariate analysis		Multivariate analysis	
	OR (95% CI)	P-value	aOR (95% CI)	P-value
Gender of household head				
Male	1		1	
Female	0.15 (0.04–0.52)	**0.003**	0.16 (0.04–0.60)	**0.006**
LLIN use				
Yes	1		1	
No	2.70 (1.59–4.59)	**0.000**	2.20 (1.22–3.98)	**0.009**
Sleep outdoors				
Yes	1		1	
No	5.71 (2.76–11.83)	**0.000**	3.79 (1.64–8.77)	**0.002**
Stagnant water outside the house				
Yes	1		1	
No	0.39 (0.23–0.65)	**0.000**	0.46 (0.26–0.82)	**0.009**

Analysis of factors associated with malaria was adjusted by participant gender, occupation of the participant, age and gender of the household head, wealth index, LLIN use, outdoor sleeping, presence of stagnant water outside the house, presence of bushes/overgrown vegetation outside the house. Goodness of fit test: Hosmer–Lemeshow, chi^2^(4) = 11.11, p = 0.0253. BIC = − 994.099, AIC = 1.222, AUC = 0.7253

Bold values indicate significant associations

Other factors, including gender of adolescents, age and level of education of the household head, wealth index, and type of house were not significantly associated with clinical malaria among study participants.

## Discussion

Malaria is an important public health problem in Senegal, with a shift of the disease burden from younger to older children and adolescents. The present study aimed at identifying factors associated with clinical malaria among adolescents aged 10–19 years, living in areas with persistent malaria transmission, to guide future interventions.

The results showed that adolescents who reported not sleeping under a LLIN are more at risk of malaria compared to those who reported LLIN usage. This result is consistent with the findings from other studies [16, 18, 28]. In Senegal as in most countries, health systems and services are mainly designed for either young children or adults [29] and, therefore, not adapted to the specific needs of adolescents. For instance, the routine distribution of LLINs at the health facilities and community levels is mainly targeting pregnant women and children under 5 years old. This situation may hinder their access to health services and information, and justifies the need for implementing adolescent-friendly integrated programmes [29, 30]. Such strategies will help to overcome adolescent health-related issues, including access to and utilization of malaria services. The NMCP could explore alternative ways of increasing the target to adolescents. This could improve coverage and utilization of preventive measures among this specific population. Other factors including belief, stigma and adolescent knowledge could also restrict their access to and use of health care services [27, 31–33]. This problem calls for the need to increase adolescent awareness with regard to malaria control and prevention. This could be achieved through school-based delivery of a package of interventions targeting both conventional and traditional schools such as the koranic schools (‘Dara’ in Senegal). It is well established that school-based health education is an effective means of sustaining health knowledge and passing this knowledge to communities [34–36]. Other places/organizations that adolescents may attend such as vocational training centres, community-based networks/organizations and youth movements/associations could also be targeted to reach out-of-school adolescents [37]. The empowerment of adolescents through a community-based peer education approach could also be an alternative to improving their awareness and knowledge of malaria prevention and promoting LLIN use among their peers. Peer education has been shown to be an effective tool for health promotion and influences positive behaviour [38–40]. It could be an additional strategy to consider by the Senegalese NMCP to reach specific groups such as adolescents.

Non-use of other preventive measures was associated with an increased risk of malaria, specifically among 10–14 years-old adolescents. In this study, other control measures included aerosol spraying, mosquito coils, weeding, IRS, sewage evacuation, and traditional methods. Most of the study participants reported the use of aerosol spraying, and the frequency of aerosol usage was higher among controls. Other studies have shown that the use of mosquito control measures, including aerosol sprays, herbs and mosquito coils is associated with a lower risk of clinical malaria [41, 42]. However, a Cochrane systematic review has not shown evidence of an association between spatial repellents such as mosquito coils and the risk of malaria [43].

Several studies have reported an association between higher risk of malaria and outdoor sleeping [1, 44]. In contrast to these findings, in this study adolescents who reported not sleeping outdoors (e.g., indoor sleeping) have a 4.74-fold higher risk of clinical malaria compared to those that reported sleeping outdoors. This result is in line with findings from a study conducted in Myanmar among migrants in gold mining, rubber and palm oil plantation areas [45]. Mosquito behaviour in this study area could explain this result. Entomological surveillance data showed that *An. gambiae s.l.* is the most predominant vector in this area, with a significantly higher indoor biting (endophagic) rate [46]. Although it is proven that LLIN is effective on endophagic vectors [47], participants might be bitten indoors when they are awake, before the use of LLINs during sleeping time. To ensure maximum protective effect against malaria, high-quality LLINs are required [48]. The increased risk of clinical malaria among children who reported indoor sleeping as the main behaviour could also be due to poor LLIN quality, but the study did not assess LLIN quality across the different households.

Adolescents aged 10–14 years old were at higher risk of malaria compared to their counterparts of 15–19 years old. In the study area, as in other southern regions in Senegal where malaria is highly seasonal and transmission important, 3 months to 10 years-old children were receiving seasonal malaria chemoprevention (SMC) with sulfadoxine-pyrimethamine and amodiaquine (SPAQ) for three to four months during the transmission season. SMC policy has been implemented in these locations since 2013 with an average coverage of full treatment courses around 80% per year [3, 5]. Thus, younger children enrolled in this study had received several rounds of SMC. Although SMC has been proven to be efficacious in reducing malaria morbidity and mortality [7, 49, 50], the strategy has the potential to induce a reduction in the malaria antibodies [51, 52]. Therefore, the increased risk of clinical malaria among 10–14 years-old children could be related to an increased susceptibility due to reduced antibody levels after several rounds of SMC. Moreover, though the SMC programme has overall strongly positive health effects, a shift of morbidity in older children can be induced by the programme if transmission levels remain static and not reduced by other interventions [53]. As the malaria burden is shifting in these areas with older children becoming more at risk, increasing SMC target up to 15 years should be evaluated as an alternative option for controlling malaria in areas with persistent malaria transmission.

The absence of stagnant water in the vicinity of the house was protective against malaria in the study participants. This finding is in line with those of other studies [19, 44, 54, 55]. This could be explained by the fact that stagnant waters are potential breeding sites of *Anopheles* mosquitoes. Therefore, having such water around the house could increase the population of mosquitoes and facilitate malaria transmission [56].

Compared to those that have males as household heads, participants who have female household heads were protected against malaria in the study and this result is specific to older adolescents, aged 15–19 years. Similar to these findings, a study conducted in India reported that households with male heads have a greater risk of malaria [57]. It is well established that in many African contexts, women usually play a primary role of care-giving to other members of the household [58], and are more committed to promoting disease prevention strategies, such as LLIN usage within their own families, as shown in a study conducted in Nampula Province, in Mozambique [59]. In addition, women are often responsible for hanging, washing and making nets usable [59].

This study also showed reduced risk of clinical malaria among apprentice and *talibé* (children in placement in Koranic school) compared to children attending classic school, particularly among 10–14 years-old children. In this study, most of the apprentices were controls and reported high LLIN use, which may explain their reduced risk of getting malaria. In this study, apprentice includes tailor, driver, mechanic, mason, and is not an occupation associated with high risk of malaria such as gold mining or farming, as shown in other studies [16, 57, 60]. Being *talibé* is also associated with a reduced risk of malaria. This result is surprising since these children are considered a vulnerable population and are at higher risk of infectious diseases such as malaria [61]. Most of the *talibé* were controls and reported use of LLINs; this could explain this result. Serological studies assessing cumulative exposure to malaria parasites would provide more insights on malaria distribution among the group of *talibé*.

## Limitation of the study

The main limitation of this study is the lack of entomological surveys to assess mosquito vectors and their biting behaviour as well as transmission intensity in the study area. However, an entomological survey conducted in 2019 provided additional information and showed that malaria vector species were predominantly represented by *An. gambiae s.l.* with an important fraction of indoor biting population [46].

In this study, the identification of cases and controls relied on RDT results. While this method is not the gold standard for malaria diagnostic, RDT results are used to define malaria episodes, according to national guidelines and in many healthcare settings, treatment practices as well as the routine surveillance system.

## Conclusion

The study revealed that environmental factors and the non-use of preventive measures are the main determinants of malaria risk among adolescents living in areas with a persistent transmission in Senegal. Strategies aiming to increase disease awareness and access to healthcare interventions, such as LLINs, are needed to improve malaria control and prevention among these vulnerable groups. This could be achieved through adolescent-friendly health programmes. In addition, extending the target of LLINs routine distribution to adolescents and increasing the SMC target to 15 years of age might be an alternative option for accelerating malaria control in these areas with persistent malaria transmission.

## Data Availability

The datasets used for the current study are available from the corresponding author on reasonable request.

## Abbreviations

NMCP: National Malaria Control Programme
RDT: Rapid diagnostic test
aOR: Adjusted odds ratio
SSA: Sub-Saharan Africa
LLIN: Long-lasting insecticide net
REDCap: Research Electronic Data Capture
DHS: Demographic and Health Survey
AIC: Akaike Information Criterion
BIC: Bayesian Information Criterion
AUC: Area under curve
SMC: Seasonal malaria chemoprevention
SPAQ: Sulfadoxine-pyrimethamine and amodiaquine
IRS: Indoor residual spraying
ACT: Artemisinin-based combination therapy
IPTp: Intermittent preventive treatment for pregnant women
DSDOM: Dispensateur de soins à domicile
PCA: Principal component analysis
